# Ecological adaptations influence the susceptibility of plants in the genus *Zantedeschia* to soft rot *Pectobacterium* spp.

**DOI:** 10.1038/s41438-020-00446-2

**Published:** 2021-01-01

**Authors:** Yelena Guttman, Janak Raj Joshi, Nofar Chriker, Nirmal Khadka, Maya Kleiman, Noam Reznik, Zunzheng Wei, Zohar Kerem, Iris Yedidia

**Affiliations:** 1 The Robert H. Smith Faculty of Agriculture, Food and Environment, The Hebrew University of Jerusalem, Rehovot, Israel; 2 Institute of Plant Sciences, Agricultural Research Organization, Volcani Center, Rishon Lezion, Israel; 3 Department of Horticulture and Landscape Architecture, Colorado State University, Fort Collins, CO USA

**Keywords:** Biotic, Natural variation in plants, Plant physiology

## Abstract

Soft rot disease caused by *Pectobacterium* spp. is responsible for severe agricultural losses in potato, vegetables, and ornamentals. The genus *Zantedeschia* includes two botanical groups of tuberous ornamental flowers that are highly susceptible to the disease. Previous studies revealed that *Z. aethiopica*, a member of the section *Zantedeschia*, is significantly more resistant to *Pectobacterium* spp. than members of the same genus that belong to the section *Aestivae*. During early infection, we found different patterns of bacterial colonization on leaves of hosts belonging to the different sections. Similar patterns of bacterial colonization were observed on polydimethylsiloxane (PDMS) artificial inert replicas of leaf surfaces. The replicas confirmed the physical effect of leaf texture, in addition to a biochemical plant–bacterium interaction. The differential patterns may be associated with the greater roughness of the abaxial leaf surfaces of *Aestivae* group that have evolutionarily adapted to mountainous environments, as compared to *Zantedeschia* group species that have adapted to warm, marshy environments. Transverse leaf sections also revealed compact aerenchyma and reduced the total volume of leaf tissue air spaces in *Aestivae* members. Finally, an analysis of defense marker genes revealed differential expression patterns in response to infection, with significantly higher levels of lipoxygenase 2 (*lox*2) and phenylalanine ammonia lyase (*pal*) observed in the more resistant *Z. aethiopica*, suggesting greater activation of induced systemic resistance (ISR) mechanisms in this group. The use of *Zantedeschia* as a model plant sheds light on how natural ecological adaptations may underlay resistance to bacterial soft rot in cultivated agricultural environments.

## Introduction

*Zantedeschia*, commonly known as calla lily, is one of the world’s most iconic ornamental plants. It is endemic to southern Africa, but has been introduced worldwide as a cut flower, pot plant, garden, or landscape plant. The flower has been developed, for a variety of colors, mainly in New Zealand, the Netherlands, and the United States^1^. *Zantedeschia* is a genus of the Araceae family, with eight species in two sections: section *Zantedeschia* with two white species (*Z. aethiopica* Spreng. and *Z. odorata* Perry.) that flower during late winter/spring, with preference to warmer-temperature marshy habitats, and section *Aestivae* with six species of mostly colored, summer flowering callas that inhabit cool-temperature mountainous environments, and exhibit complete winter senescence (*Z. albomaculata* Baill., *Z. elliottiana* Engl.*, Z. jucunda* Letty., *Z. pentlandii* Wittm.*, Z. rehmannii* Engl., and *Z. valida* Singh)^2–4^. Calla lilies are easily propagated through vegetative reproduction, yet, further horticultural development is limited due to high sensitivity to bacterial soft rot and virus infections^5^.

Soft rot disease in *Zantedeschia* is caused mainly by *Pectobacterium carotovorum*, *P. aroidearum*, and *Dickeya dadantii*, former members of the Erwinia group^6,7^*. P. brasiliense* (Pb) is an emerging pathogen in the family Pectobacteriaceae that has been reported in a wide range of hosts, including ornamental plants and vegetables such as *Solanum tuberosum* (potato), *S. lycopersicum* (tomato), and *Cucumis sativus* (cucumber)^8^. Pb tolerance to a wide range of temperatures, promoted its dispersal to different climate zones, from tropical regions such as Brazil to temperate regions such as Europe and North America^9^. *Pectobacterium* spp. are Gram-negative bacteria that utilize synchronized production of plant cell wall-degrading enzymes (PCWDEs) as their main virulence attribute^8,10,11^, and enter the host through stomatal openings and wounds, colonize xylem vessels, parenchyma, and protoxylem cells^12–14^. Once temperature, humidity, and pH are suitable, the bacteria proliferate up to a critical number and start producing and secreting large amounts of PCWDEs that ultimately macerate the plant tissue^15^. They attack aboveground plants or underground storage organs, both in the field and in the warehouse, causing severe losses^16^.

Studies have characterized differences in the susceptibility of the two *Zantedeschia* sections to *P. carotovorum* infections^2–4^. None have suggested a mechanism that underlies the observed variability. With no curative measures for the disease, integration of disease-resistant cultivars into breeding programs may become a viable tool to overcome the disease. However, although the evergreen *Z. aethiopica* is more resistant to *Pectobacterium* infection than the winter-dormant cultivars, the two groups cannot be crossed^17,18^. Interspecific hybrids suffer from plastome–genome incompatibility, producing degenerated, chlorophyll-deficient abnormal embryos^4,16,18^.

Here, we have characterized morphological and biochemical differences that may explain the higher resistance of the section *Zantedeschia* to bacterial infection: anatomical differences and physical barriers that limit bacterial colonization, and immune response and activation of defense-related genes to combat bacteria^19^. These traits are suggested to result from the distinct ecological pressures, both biotic and abiotic, in the natural habitat of each *Zantedeschia* group. Ultimately, resolving the protecting features in the resistant *Zantedeschia* should open new paths for breeding toward increased tolerance and even resistance against soft rot disease.

## Results

### Symptoms and biochemical reactions of infected *Zantedeschia* varieties

**Fig. 1:**
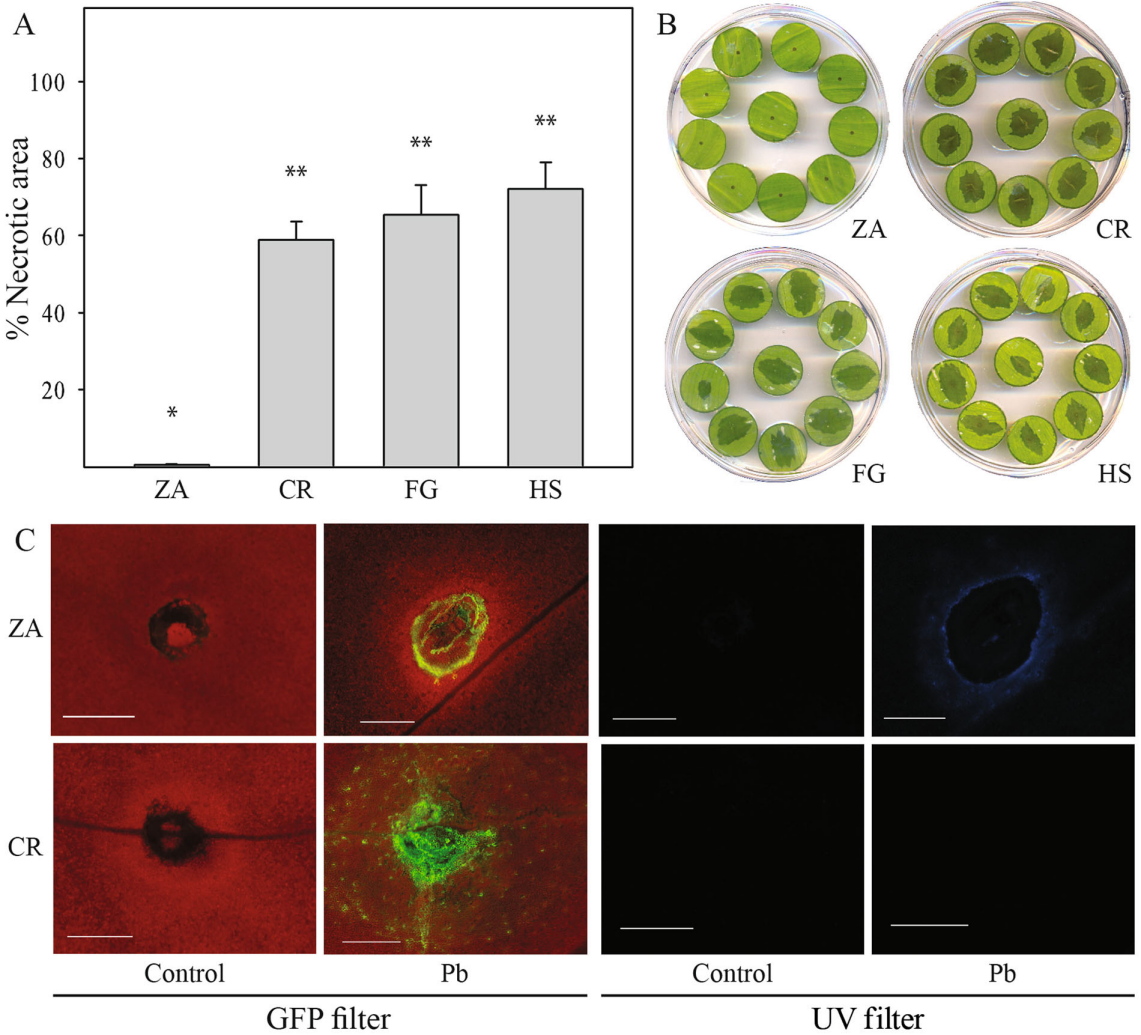
Soft rot symptoms on *Zantedeschia* (calla lily) leaf discs taken from different cultivars and infected with *Pectobacterium brasiliense* (Pb): *Z. aethiopica*, ZA; “Captain Romance”, CR; “Florex Gold”, FG; “Hot Shot”, HS. **A** Symptoms were determined as the percentage of macerated tissue out of total leaf disc area, 24 h post inoculation with 10 µl of bacterial suspension (10^8^ CFU/ml, OD_600_ = 0.1), and incubation at 28 °C. Data represent means ± SE of three independent experiments with four plates taken from different plants containing ten replicates for each cultivar. Treatments labeled differently are significantly different (*P* < 0.05). **B** Representative pictures of one plate of each of the tested cultivars. **C** Confocal images of ZA and CR inoculated as above with Pb expressing GFP or with distilled water (control). The point of inoculation was viewed under a fluorescent microscope with a GFP filter (left) and a UV filter (right), 24 h post inoculation

Susceptibility of calla lilies to soft rot infection was studied using leaf disc assays, measuring the development of necrotic area following infection^14,16^. The results revealed significant variability in the progress of disease between white *Zantedeschia* (ZA), displaying less necrotic area and the commercial colored hybrids (*Aestivae*) “Florex Gold” (FG, yellow), “Captain Romance” (CR, pink), and “Hot Shot” (HS, orange) (Fig. 1A, B). CR, a colored representative hybrid, was used for infection assays with a GFP-labeled *P. brasiliense* strain (Pb + ) that allowed monitoring bacterial colonization of tissues 24 h post inoculation. Confocal microscopy images revealed denser and more intense colonization around the inoculation site of CR in comparison to ZA. Moreover, using UV excitation, clear fluorescent rings around the bacterial penetration sites were observed in ZA but not in CR (Fig. 1C). Such fluorescence is typical of the accumulation of phenolic compounds, during plant defense responses and is often accompanied by upregulation of oxidizing enzymes, mainly peroxidases (PODs) and polyphenol oxidases (PPOs). POD and PPO analysis in both *Zantedeschia* groups revealed higher basal levels of POD activity in CR, as compared with ZA (Supplementary Fig. S1); however, following infection with Pb, POD activity was downregulated in CR, while in ZA, similar activity levels were observed in both treated and control samples. PPO activity increased significantly in ZA upon inoculation with Pb (Supplementary Fig. S1), and not in CR.

### Differential leaf morphology

**Fig. 2:**
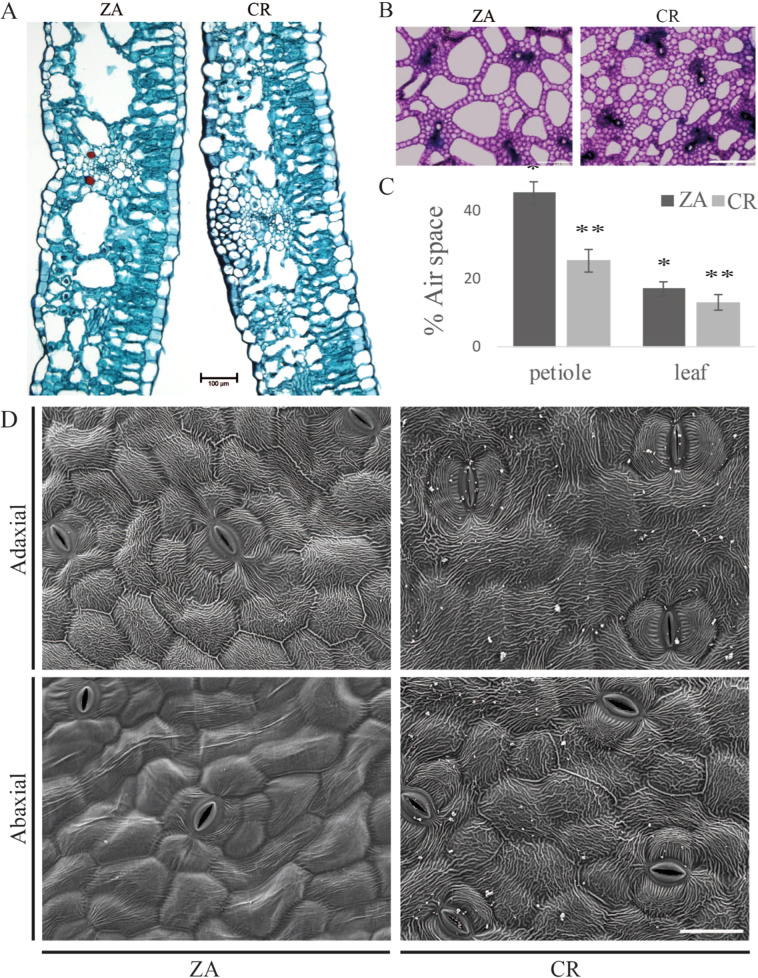
Microscopic examination of leaf and petiole morphology of *Zantedeschia aethiopica*, (ZA) and the colored hybrid “Captain Romance” (CR). **A** Transverse leaf section of ZA and the hybrid CR stained with safranin-fast-green viewed under a light microscope. Resolution ×10, black bar 100 μm. **B** Transverse petiole section of ZA and the hybrid CR stained with toluidine blue and viewed under a light microscope. Resolution ×5, white bar 500 μm. **C** Air-space ratio was calculated using ImageJ software, as a percentage of section area. Data represent means ± SD of six fully opened second leaves. Treatments labeled with * are significantly different (*P* < 0.01). **D** Scanning electron microscopy (SEM) images of adaxial (upper) and abaxial (lower) leaf surfaces of ZA and CR. Resolution ×400, white bar 50 μm

Transverse leaf sections of CR and ZA, representing the two calla lily groups, were stained with safranin-fast green and viewed under a light microscope. Lower tissue compactness and higher content of air spaces were observed in the mesophyll tissue of ZA than of CR (Fig. 2A). A similar pattern was observed in transverse petiole sections stained with toluidine blue (Fig. 2B). Quantitative determination of the accumulated air space per section area indicated far higher air-space volumes in leaves and petioles of ZA than of CR (Fig. 2C).

Adaxial (upper) and abaxial (lower) leaf surfaces were observed using a scanning electron microscope (SEM). The adaxial leaf surface was covered with a cuticle that was not different between ZA and CR. However, the abaxial surface of the leaves was clearly different between the two groups, displaying smooth surfaces for ZA and ridged rough pattern for CR (Fig. 2D).

### Bacterial colonization of leaf surfaces

**Fig. 3:**
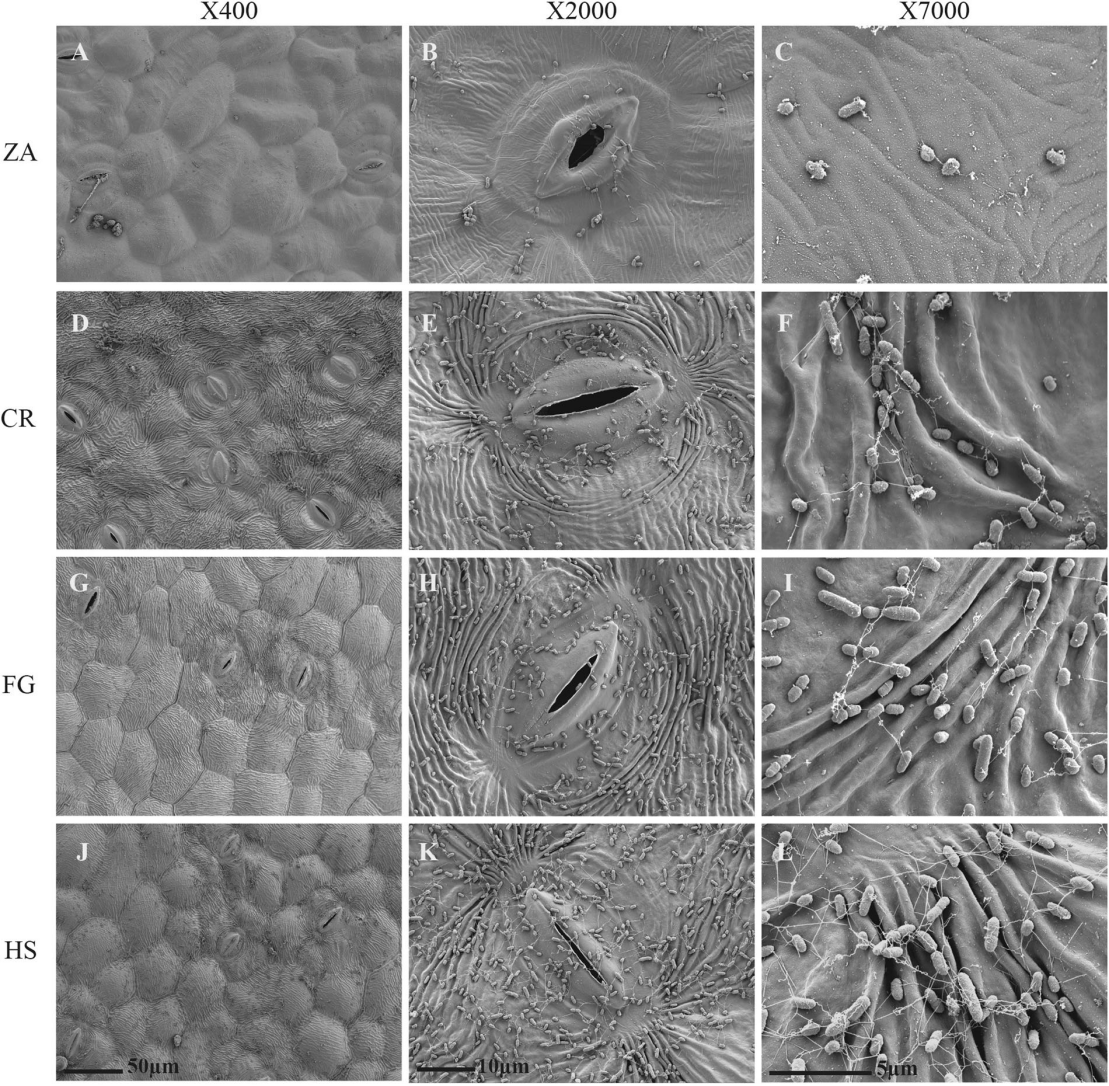
Scanning electron microscopy (SEM) of the abaxial (lower) leaf surface of *Zantedeschia cultivars*. *Z. aethiopica*, ZA; “Captain Romance”, CR; “Florex Gold”, FG; “Hot Shot”, HS. The photos were taken 3 h post application of 10 µl of *Pectobacterium brasiliense* (Pb) suspension (10^8^ CFU/ml, OD_600_ = 0.1) onto the leaf surface. The leaves were allowed to dry for 3 h at room temperature in a laminar flow hood, before fixation in 70% ethanol overnight, followed by dehydration with 90, 95, and 100% ethanol. The scale for each image is given in µm, and the resolution is designated at the top

Bacterial colonization varied greatly between the two *Zantedeschia* cultivars, in line with the differences in leaf surface patterns. Dense colonization of *Pectobacterium* cells was observed on the abaxial leaf surface of CR using SEM, and only scattered cells on ZA (Fig. 3). Biofilm formation and exocellular polymers, observed in higher-resolution scans, followed the same trends. Submicrometer-scale appendages were observed on the leaf surface of the infected CR and not on ZA. Ridges and grooves, typical to the surface of the colored hybrids, supported bacterial establishment, with the orange hybrid HS being the most densely colonized.

**Fig. 4:**
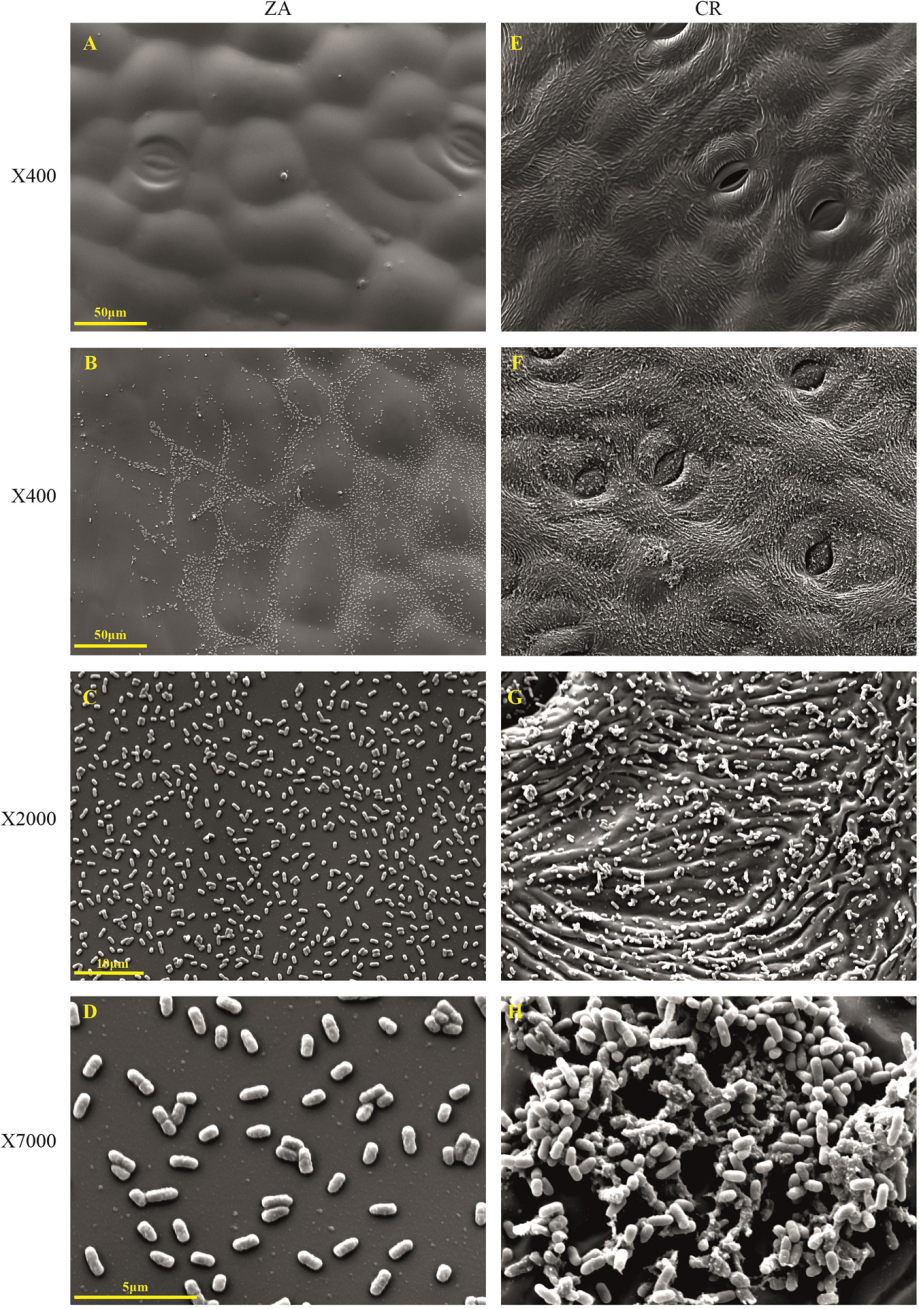
Polydimethylsiloxane (PDMS) artificial leaf surfaces of the abaxial side of *Zantedeschia aethiopica* (ZA) and the hybrid “Captain Romance” (CR) following application of *Pectobacterium brasiliense* (Pb). In total, 10 µl of bacterial suspension (10^8^ CFU/ml, OD_600_ = 0.1) were applied onto the surface of the replicas. **A** PDMS replica of ZA abaxial leaf surface. **B**–**D** ZA artificial surface following Pb application at a resolution of ×400, ×2000, and ×7000. **E** PDMS replica of CR as above. **F**–**H** Higher-resolution images of CR following bacterial application as above

To further test if differences in the pattern of the leaf surface could underlie group behavior and colonization by Pb, artificial leaf surfaces were produced using silicon-based polymer PDMS, based on both ZA and CR. The artificial surfaces, chemically identical and structurally different, revealed distinctive colonization patterns that were strictly dependent on the architecture of the leaf surface. Three hours after Pb application to the surface, the smoother pattern of ZA was covered with bacterial cells that were apparently washed into lower topographic grooves on the surface. The bacterial cells were dispersed individually on the leaf plane (Fig. 4A–D); at the same time period, the artificial surface of CR, encouraged bacterial cell to attach and colonize the rigid, notched leaf plane already producing small cell clusters gathering into established biofilms (Fig. 4E–G), as observed by SEM at a higher resolution (Fig. 4H).

### Expression of plant defense-related genes

**Fig. 5:**
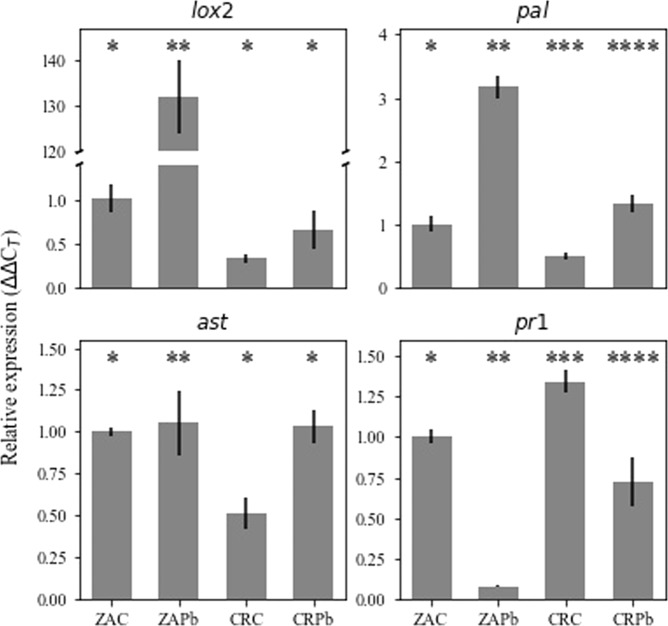
Expression levels of defense-related genes in *Zantedeschia aethiopica* (ZA) or “Captain Romance” (CR) in response to *Pectobacterium brasiliense* (Pb) inoculation. Leaf inoculation was made with 100 µl of bacterial suspension (10^6^ CFU/ml, OD_600_ = 0.001) or distilled water (control, C). Relative transcripts levels were determined for *lox*2, *ast*, *pal*, and *pr*1, and normalized to *tubulin*. Bars represent the mean relative expression ± SD of two independent experiments with three replicates. Bars not labeled by the same mark indicate a significant difference (*P* < 0.05)

Changes in the expression of representative plant defense genes were studied to further elucidate the difference in the response of ZA and CR to Pb inoculation (Fig. 5). Lipoxygenase 2 (*lox*2), phenylalanine ammonia lyase (*pal*), aspartate aminotransferase (*ast*), and pathogenesis-related protein (*pr*1) were cloned, and primers were designed for a qRT-PCR protocol (Table 1) to allow expression analysis. Basal levels of *lox2*, *pal*, and *ast*, representing the ISR signaling pathway, were lower in CR relative to ZA, under control conditions. In response to inoculation with Pb, the expression of *lox*2 and *pal* markedly increased in ZA, but only *pal* increased in CR. These results suggest the induction of the ISR signaling pathway in both cultivars, with a stronger response of ZA. The representatives of the SAR signaling pathway *pr*1 were downregulated in both plants following Pb inoculation.

**Table 1:** Primers used for defense-related genes expression assay

A		
Gene name	Primers (5’ → 3’)	Estimated product size
Lipoxygenase 2 (*lox*2)	F: CAA TGA CTT GGG TAA TCC AGA	950
	R: CAT GAG TTC TCA ACC AGT GG	
Phenylalanine ammonia lyase (*pal*)	F: ATC AAC ACC CTC CTC CAG GGG TA	670
	R: CGG GTT GTC GTT CAC GGA GTT GA	
Aspartate aminotransferase (*ast*)	F: GTG CCC ACA ACC CTA CTG G	410
	R: CAG CCA TGC CCT TCA GCT C	
Pathogenesis-related protein (*pr*1)	F: AAA GCT CAA GAT AGC CCA CAA G	410
	R: GGC TTC TCG TTC ACA TAA TTC C	

B		
Gene name	Primers (5’ → 3’)	Product size
Lipoxygenase 2 (*lox*2)	F: CAT CAA GCT GCC AAG AGG TT	118
	R: GCA ACC AAG AAA ATC CGT CT	
Phenylalanine ammonia lyase (*pal*)	F: GAC CTC GTC CCG CTC TCC TAC A	96
	R: CTC CAC AGC AGA GAC GTG GTG AC	
Aspartate aminotransferase (*ast*)	F: GCC AGT GGT GAT CCT GAG AG	133
	R: GGA TGC TGA GGC AAC CTA CT	
Pathogenesis-related protein (*pr*1)	F: GGT AGA ACC TCT TCT GGG GAT G	99
	R: AGT TGC TTC GGT AGT CGT AGT AC	
Actin	F: GAC TCA AAT CAT GTT AGA GAC ATT CAA	114
	R: GTA CGG CCA CTG GCA TAG A	

(A) Primers used for sequencing of conserved sites of defense-related genes. Primers were designed based on the alignment of cDNA sequences of the monocot species *Zea mays*, *Triticum aestivum*, *Oryza sativa*, and of the dicot model plant *Arabidopsis thaliana*. (B) Primers used for qRT-PCR

## Discussion

The genus *Zantedeschia* is divided into two botanical sections: the section *Zantedeschia* with *Z. aethiopica* (white), being relatively tolerant to bacterial soft rot, and section *Aestivae* (colored hybrids) being highly susceptible^2,17^. Soft rot in the genus *Zantedeschia* is caused mainly by *Pectobacterium* spp. through all phases of field production and storage^20^. We hypothesized that differences in the response of the two *Zantedeschia* groups to *Pectobacterium* infection may illuminate traits that are associated with higher resistance to the bacterium. Previous studies have characterized *Zantedeschia* accessions with relatively higher resistance to *P. carotovorum*^16,17,21^, suggesting a genetic background for soft rot resistance based on heredity patterns. The traits underlining these patterns remain unclear^2^. Using *P. brasiliense* (Pb), an emerging soft rot agent in warmer climate regions in potato and ornamentals^22^, our results confirmed earlier studies with *P. carotovorum* (formerly *Erwinia carotovora* subsp. *carotovora*) showing that colored *Aestivae* hybrids (FG, CR, and HS) are more susceptible to Pb infection than ZA, as they all developed typical soft rot disease symptoms with large necrotic areas^2,4,17^.

The plant canopy is a major factor in environmental survival, establishment, and spread of bacterial diseases. Accordingly, we have focused on leaf architecture, which is the first layer for bacterial attachment, colonization, and penetration. Transverse sections of leaves and petioles allowed the characterization of the in-planta environment. Surprisingly, major differences in leaf surface textures between the two *Zantedeschia* groups were observed. The smoother, less complex architecture abaxial surface of ZA differed significantly from that of CR (and other colored hybrids). On the other hand, transverse sections displayed larger air spaces occupying more parenchyma space in ZA compared to the more compact tissue of CR. Even more pronounced is the difference in the air spaces in petioles, almost twofold higher in ZA than in CR. We speculate that the differences may have resulted from ecological niche adaptations of the two botanical sections. Though both originated from southern Africa (Cape province, Lesotho, Natal, Swaziland), ZA is almost exclusively confined to the southeastern coastal belt at altitudes up to 1000 m of marshy wetlands; the colored *Zantedeschia* is restricted to the mountainous regions at altitudes of above 1200 and up to 2000 m^1^. The morphological features of smoother leaf surfaces and larger air spaces of the aerenchyma in leaves and petioles are part of habitat adaptation associated with wetlands and marshy valleys of lower altitude, inhabited by ZA^23^, while *Aestivae* species favor well-drained soil, with more condensed tissue and compact life form, typical of higher altitude. These features may also impact oxygen availability and possibly earlier development of anaerobic conditions during a bacterial attack. Anaerobiosis may affect oxygen-dependent host defenses, cell wall lignification, and suberization on the one hand, but also bacterial virulence and production of pectic enzymes on the other^24,25^. Larger air spaces may thus determine bacterial compatibility and contribute to higher resistance of ZA. SEM micrographs taken as soon as 3 h post bacterial application to the leaf surfaces showed massive colonization on the colored hybrids (CR, FG, and HS) with structures typical of bacterial group behavior. In contrast, lower cell densities with infrequent colonies were observed on ZA. Since soft rot dispersal in agricultural systems is often associated with irrigation water or aerosols^26^, it is obvious that leaf surfaces facing the ground have a significant impact on bacterial attachment. After successful attachment, pioneering bacteria penetrate the leaf through stomatal openings, hydathodes, or wounds, colonize the intercellular spaces, and move forward to other tissues through the vascular system^3,13,27^. To independently study the effect of leaf architecture on bacterial attachment and colonization, an artificial polymer replica of leaf surfaces was constructed by PDMS. Colonization patterns were recorded 4 h post bacterial application to the artificial surface. The results revealed comparable patterns to those observed on true leaf surfaces. The effects of surface architecture on the formation of biofilms have been previously reported, using artificial nanostructure features^28^. Surface topography also affected biofilm development when the mesoscale-patterned polymer was used, influencing species colonization, growth, and persistence when exposed to antibiotics^29^. Here, leaf architecture was at least in part responsible for providing better conditions to *Pectobacterium* establishment on the colored *Zantedeschia* as observed by the formation of submicrometer-scale appendages similar to those observed in *E. coli* during cell adhesion and biofilm formation^30^. Other factors that influence leaf susceptibility/tolerance to soft rot have been previously shown to involve antimicrobial compounds, as well as innate or induced defense mechanisms^14,31,32^. Defenses against *Pectobacterium* spp. involve both SAR and ISR signaling pathways^19^. Here, limited fluorescence of GFP-labeled Pb+ cells was observed on ZA following infection, which was accompanied by the accumulation of phenolic compounds around the penetration site (observed under UV filter). The accumulation of phenolic compounds around the inoculation site could be a part of the plant defense response^33^. The antimicrobial properties of phenolic compounds^34,35^ could have resulted in restricted growth in ZA. Phenolic accumulation in ZA co-occurred with higher activity of PPO, the enzyme that oxidizes phenolics to quinones, which are suggested to be involved in plant defense against microbial pathogens^36^. Expression of defense-related genes lipoxygenase 2 (*lox*2), aspartate aminotransferase (*ast*), pathogenesis-related protein (*pr1*), and phenylalanine ammonia lyase (*pal*) was studied. *lox2* encodes a key enzyme in the biosynthesis of the defense-signaling hormone jasmonic acid and is a representative of the ISR pathway in plants^37^. The largest increase in its expression was observed in ZA following Pb inoculation, which supports the induction of ISR signaling pathway in this section. *ast* is a regulator of carbon–nitrogen metabolism and amino acid synthesis during plant defense response to necrotrophs^38^. Higher expression of *ast* in Arabidopsis was correlated with increased sensitivity to *Botrytis cinera*^39^. Here, *ast* expression was upregulated in CR response to Pb infection, while in ZA, its expression was unchanged upon bacterial infection. Similarly, expression of *pr1*, the most recognized marker of SAR^40^, was downregulated in both *Zantedeschia* cultivars in response to Pb inoculation, supporting previous reports showing that SAR is less efficient in resistance to *Pectobacterium* spp^14,33^. Finally, *pal* expression is recognized as the first committed step in the phenylpropanoid pathway, leading to the synthesis of phenolic compounds. Expression levels of this gene were increased in both *Zantedeschia* sections, with a stronger response in ZA, confirming its involvement in resistance to *Pectobacterium* spp^36^. This observation fits with the accumulation of phenolic compounds around the infection site in ZA.

In conclusion, the involvement of less explored aspects of host–plant interactions with the soft rot bacterium Pb was demonstrated. The emphasis on plant morphological features and environmental adaptations revealed higher resistance of *Zantedeschia* section to *Pectobacterium* spp. infection. This resistance mechanism is a multifaceted phenomenon that involves several factors of which surface area architectures and tissue compactness play a role in addition to differential induced defense responses following pathogen infection.

## Materials and methods

### Plant material, bacterial strains, and growth conditions

Commercially grown calla lily plants: white calla lily (*Zantedeschia aetheopica*) ZA and colored calla lily hybrids (“Captain Romance”, CR; “Florex Gold”, FG; “Hot Shot”, HS) were grown in a greenhouse (25/10 °C maximum/minimum, natural daylight). The youngest fully spread leaf was cut at the base of the petiole and used fresh for all analyses. Pb isolated from potato and the same strain carrying green fluorescence protein—GFP (Pb + ) were used in the study. The strains were cultured at 28 °C in Luria–Bertani (LB) medium (Difco Laboratories, MI, USA), supplemented with ampicillin 100 µg/ml for Pb + , under continuous shaking of 200 rpm. *P. brasiliense* has a broad temperature range—20–39 °C with optimum at 31–32 °C^9^. In all, 28 °C is a suboptimal temperature for *P. brasiliense* and was chosen to avoid drastic temperature change to the plants grown at 25 °C and to slow down the rapid response of the highly sensitive colored hybrids.

### Screening for resistance to soft rot infection

Leaf discs excised from different calla lily cultivars were prepared as previously described^14^, kept on half-strength Murashige and Skoog (MS) minimal medium, and challenged with 10 µl of bacterial suspension (10^8^ CFU/ml, OD_600_ = 0.1) of Pb or distilled water as a control. The inoculated plant material was incubated at 28 °C. Disease progress was evaluated 24 h post inoculation, as the percentage of tissue decay relative to the total area of the leaf disc. The decayed area was measured using the Threshold_Colour plugin of the imageJ software (NIH, MD, USA). Three independent experiments were carried out, each using four leaves, with 10 replicates, 40 leaf discs per treatment (Pb/control for every cultivar). Fluorescence microscopy observations of the inoculated leaf discs were conducted as previously described^33^.

### Microscopic examination

Transverse hand sections cut from the petiole were put in distilled water and stained with toluidine blue as described by Villodron^41^. Also, leaf sections were prepared as explained by Ruzin et al.^42^. In brief, small pieces of leaves were fixed with FAA (formalin: glacial acetic acid:ethyl alcohol: 5:5:90). Fixation was followed by an ethanol dilution series and subsequent stepwise exchange of ethanol with ‘Histoclear’ (xylem substitute, National Diagnostics GA, USA). Samples were then embedded in paraffin and cut with an RM2245 microtome (Leica Biosystems, Germany) into 20-μm thick sections. Furthermore, sections were stained using safranin-fast green and examined with a Leica DMLB microscope (Leica Microsystems, Germany) to observe the tissue morphology. The microscope was equipped with a DS-Fi1 camera (Nikon Instruments Inc., Japan).

SEM observations of the leaf surface and attachment of the bacteria to the abaxial leaf surface of different cultivars were studied 3 h post application of 10 µl of bacterial suspension (10^8^ CFU/ml, OD_600_ = 0.1) onto the leaf surface. The leaves were allowed to dry for 3 h before fixation of the samples in 70% ethanol overnight, followed by dehydration with 90%, 95%, and 100% ethanol, each for an hour. Finally, samples were dried on K850 critical point dried and coated with gold–palladium alloy on mini sputter coater, following the manufacturer’s instructions (Quorum Technology Ltd., UK). The samples were observed under SEM, Jeol JSM 5410 (JEOL Inc, MA, USA). Leaves for SEM were taken from three different plants of each cultivar, with ten replicates each. Images are representative of bacterial colonization patterns on each of the calla lily cultivars/hybrids.

### PDMS-BPS surface fabrication

Polydimethylsiloxane (PDMS) prepolymer and curing agent from Sylgard™ 184 silicone elastomer kit (The Dow Chemical Company, MI, USA) were mixed at a ratio of 10:1 w/w, stirred thoroughly, and degassed under vacuum. A calla lily leaf was glued on a Petri dish with the abaxial side facing up. The PDMS was poured onto the leaf, vacuumed for 2 h, and was cured on the bench at room temperature overnight. The next day, the PDMS layer, known as the negative replica, was gently peeled off from the leaf. The negative replica was activated by exposure to plasma torch BD-20ACV, High-Frequency Generator (Electro-Technic Products, IL, USA) for 30 s. The negative replica was then placed in a desiccator with 100 µl of Trichloro (1H, 1H, 2H, 2H-perfluoro-octyl) silane (Sigma-Aldrich, MO, USA) and subjected to vacuum for 3 h. After activation, a mixture of PDMS and curing agent (10:1) was poured over a negative replica. The negative and the liquid polymer were placed under vacuum for 2 h followed by curing at RT overnight. The newly formed polymer layer, known as the positive replica, was gently separated from the negative replica and was later used for bacteria colonization assay and visualization. Three PDMS-positive replicas of both ZA and CR leaves were used for SEM study, 4 h post inoculation with 10 µl of bacterial suspension (10^8^ CFU/ml, OD_600_ = 0.1). The experiment was repeated twice.

### Luminescence and fluorescence imaging

To visualize in situ reaction 24 h post inoculation with Pb + (Pb labeled with GFP), leaf samples were examined under a Leica MZFLIII stereomicroscope (Leica Microsystems, Germany) equipped with a DS-Fi1 camera (Nikon, NY, USA) and NIS-Elements (Nikon, NY, USA) software (ver. 3.06). GFP was excited with 440–520-nm light and emission was detected through a 520–600-nm GFP2 filter. To observe phenolic compounds' autofluorescence, 320–400-nm lasers were used for excitation, and emission was assessed through a UV filter at 420 nm.

### Total RNA extraction and cDNA preparation

The total RNA was isolated from leaf segments of control and infected ZA or CR leaves, using RNA buffer (10 mM Tris-HCl, pH 8.0; 1 mM LiCl, 0.2 mM EDTA, and 1% LiDS) and hot phenol. For each cultivar, three biological replications were made per treatment, each with three replicates. Leaves were infiltrated with 100-µl bacterial suspension (10^6^ CFU/ml, OD_600_ = 0.001), applied to four spots on each leaf. Leaf tissue, 200-mg segments, 24 h after inoculation with Pb, were ground to a fine powder by mixer mill tissuelyser (Retch, Germany) under liquid nitrogen. RNA was extracted and cDNA was prepared as previously reported^43^.

### Primer design and quantitative reverse transcriptase qRT-PCR

The whole-genome sequence of calla lily is not available; thus, cDNA sequences of the monocot species *Zea mays*, *Triticum aestivum*, *Oryza sativa*, and of the dicot model plant *Arabidopsis thaliana* were aligned to identify the conserved sites across all species. Primers were designed, based on these conserved sites, and PCR was performed on the cDNA of the calla lily plant tissues (Table 1). PCR products were then sequenced in a 3730 DNA analyzer (Applied Biosystems, CA, USA) to obtain cDNA sequences of specific genes in calla lily (*Zantedeschia* sp.). These were further used to design primers for qRT-PCR (Table 1) to quantify the expression of defense-related genes in this study. PCR products of the latter primers were cloned into a pGEM-T vector using pGEM^®^-T Easy Vector System kit (Promega, WI, USA). In all, 2 µl of PCR product was mixed with 50 µl of competent *E. coli* (TOP10) DH5α on ice, then a heat shock protocol was applied at 42 °C for 45 s followed by ice. The cells were incubated on a rotary shaker for 1 h at 37 °C and then plated on a selective medium containing 100 µg ml^−1^ ampicillin, and additional 100 μl of isopropyl β-D-1-thiogalactopyranoside (IPTG) 0.1 M and 20 μl of 5-bromo-4-chloro-indolyl-β-D-galactopyranoside (X-GAL) 0.5 g ml^−1^ for a blue-white screen. Transformed colonies were grown overnight with LB supplemented with 100 µg ml^−1^ ampicillin and plasmid DNA was extracted with PureYield™ Plasmid Miniprep System (Promega, Madison, WI, USA) and sequenced for validation using the T7 primer 5’-TAATACGACTCACTATAGGG-3'^44^.

The SYBR^®^ Green (Applied Biosystems, USA) qRT-PCR assay was used to determine the expression of defense-related genes in ZA and CR following challenge infection with Pb. Real-time PCR amplifications were performed in Step One Plus real-time PCR system (Applied Biosystems, CA, USA) using gene-specific primers (Table 1), as previously reported^35^. The data were analyzed by the comparative C_T_ (ΔΔC_T_) method, with expression normalized to the expression of the reference gene actin.

### Data analysis

Data were analyzed for significance using JMP software (SAS, Cary, NC, USA) by Student’s *t* test, or ANOVA with Tukey–Kramer HSD. *P* < 0.05 was considered statistically significant unless specified otherwise.

## Supplementary information

Supplemental Material

Figure S1

## Data Availability

The dataset of gene fragments sequenced in this study is available at figshare.com (10.6084/m9.figshare.12326495).

## References

[1] Funnell, K. A. ‘Zantedeschia'. In *The Physiology of Flower Bulbs* (eds. De Hertogh, A., & Le Nard, M.) 683–704. Elsevier, 1993.

[2] Snijder RC, Cho H-R, Hendriks MMWB, Lindhout P, van Tuyl JM (2004). Genetic variation in Zantedeschia spp. (Araceae) for resistance to soft rot caused by Erwinia carotovora subsp. carotovora. Euphytica.

[3] Singh, U. S., Singh, R. P. & Kohmoto, K. *Pathogenesis and Host Specificity in Plant Diseases: Histopathological, Biochemical, Genetic, and Molecular Bases.* Pergamon, 1995*.*

[4] Wei Z (2017). Assessing genetic diversity and population differentiation of colored Calla Lily (Zantedeschia Hybrid) for an efficient breeding program. Genes.

[5] Hu W-C, Huang C-H, Lee S-C, Wu C-I, Chang Y-C (2009). Detection of four calla potyviruses by multiplex RT-PCR using nad5 mRNA as an internal control. Eur. J. Plant Pathol..

[6] Ma B (2007). Host range and molecular phylogenies of the soft rot enterobacterial genera Pectobacterium and Dickeya. Phytopathology™.

[7] Yishay M (2008). Differential pathogenicity and genetic diversity among *Pectobacterium carotovorum* ssp. carotovorum isolates from monocot and dicot hosts support early genomic divergence within this taxon. Environ. Microbiol..

[8] Charkowski AO (2018). The changing face of bacterial soft-rot diseases. Annu. Rev. Phytopathol..

[9] du Raan S, Coutinho TA, van der Waals JE (2016). Cardinal temperature differences, determined in vitro, between closely related species and subspecies of pectinolytic bacteria responsible for blackleg and soft rot on potatoes. Eur. J. Plant Pathol..

[10] Toth IK, Bell KS, Holeva MC, Birch PRJ (2003). Soft rot erwiniae: from genes to genomes. Mol. Plant Pathol..

[11] Charkowski, A. O. The soft rot Erwinia. In *Plant-Associated Bacteria* (ed. Gnanamanickam, S. S.) 423–505 (Springer Netherlands, 2006).

[12] Czajkowski R, Pérombelon MCM, Veen JAvan, Wolf JMvander (2011). Control of blackleg and tuber soft rot of potato caused by Pectobacterium and Dickeya species: a review. Plant Pathol..

[13] Kubheka GC, Coutinho TA, Moleleki N, Moleleki LN (2013). Colonization patterns of an mCherry-tagged *Pectobacterium carotovorum* subsp. brasiliense strain in potato plants. Phytopathology.

[14] Luzzatto T (2007). Efficient, long-lasting resistance against the soft rot bacterium *Pectobacterium carotovorum* in calla lily provided by the plant activator methyl jasmonate. Plant Pathol..

[15] De Boer SH (2003). Characterization of pectolytic Erwinias as Highly sophisticated pathogens of plants. Eur. J. Plant Pathol..

[16] Snijder RC, Brown FS, van Tuyl JM (2007). The role of plastome-genome incompatibility and biparental plastid inheritance in interspecific hybridization in the Genus Zantedeschia (Araceae). Floriculture Ornamental Biotechnol..

[17] Snijder RC, van Tuyl JM (2002). Evaluation of tests to determine resistance of Zantedeschia spp. (Araceae) to soft rot caused by Erwinia carotovora subsp. carotovora. Eur. J. Plant Pathol..

[18] Yao J-L, Cohen D, Rowland RE (1994). Plastid DNA inheritance and plastome-genome incompatibility in interspecific hybrids of Zantedeschia (Araceae). Theor. Appl. Genet..

[19] Davidsson PR, Kariola T, Niemi O, Palva ET (2013). Pathogenicity of and plant immunity to soft rot pectobacteria. Front. Plant Sci..

[20] Wright PJ, Burge GK, Triggs CM (2002). Effects of cessation of irrigation and time of lifting of tubers on bacterial soft rot of calla (Zantedeschia spp.) tubers. N.Z. J. Crop Horticultural Sci..

[21] Cho, H. R. et al. Virulence variation in 20 isolation of Erwinia carotovora subsp. carotovora on Zantedeschia cultivars in Korea. *Acta Horticu.* 653–659. 10.17660/ActaHortic.2005.673.90 (2005).

[22] Wolf JMvander (2017). Virulence of *Pectobacterium carotovorum* subsp. brasiliense on potato compared with that of other Pectobacterium and Dickeya species under climatic conditions prevailing in the Netherlands. Plant Pathol..

[23] Cutler, D. F., Botha, T. & Stevenson, D., Wm. Adaptive features. In *Plant Anatomy: An Applied Approach* (Blackwell publishing Ltd, 2008).

[24] Maher E, Kelman A (1983). Oxygen status of potato tuber tissue in relation to maceration by pectic enzymes of *Erwinia carotovora*. Phytopathology.

[25] Babujee L (2012). Evolution of the metabolic and regulatory networks associated with oxygen availability in two phytopathogenic enterobacteria. BMC Genomics.

[26] Perombelon MCM, Kelman A (1980). Ecology of the soft rot Erwinias. Annu. Rev. Phytopathol..

[27] Pérombelon MCM (2002). Potato diseases caused by soft rot erwinias: an overview of pathogenesis. Plant Pathol..

[28] Hochbaum AI, Aizenberg J (2010). Bacteria pattern spontaneously on periodic nanostructure arrays. Nano Lett..

[29] Bhattacharjee A, Khan M, Kleiman M, Hochbaum AI (2017). Effects of growth surface topography on bacterial signaling in coculture biofilms. ACS Appl. Mater. Interfaces.

[30] El Abed, S., Ibnsouda, S.K., Latrache, H. & Hamadi, F. Scanning electron microscopy (SEM) and environmental SEM: suitable tools for study of adhesion stage and biofilm formation. In *Scanning Electron Microscopy* (ed. Kazmiruk, V.) IntechOpen, 2012.

[31] Luzzatto-Knaan T, Kerem Z, Lipsky A, Yedidia I (2013). A systemic response of geophytes is demonstrated by patterns of protein expression and the accumulation of signal molecules in *Zantedeschia aethiopica*. Plant Physiol. Biochem..

[32] Wegener CB, Jansen G (2007). Soft-rot resistance of coloured potato cultivars (*Solanum tuberosum* L.): the role of anthocyanins. Potato Res..

[33] Luzzatto T (2007). Priming of antimicrobial phenolics during induced resistance response towards *Pectobacterium carotovorum* in the ornamental monocot Calla Lily. J. Agric. Food Chem..

[34] Joshi JR, Burdman S, Lipsky A, Yedidia I (2015). Effects of plant antimicrobial phenolic compounds on virulence of the genus Pectobacterium. Res. Microbiol..

[35] Joshi JR, Burdman S, Lipsky A, Yariv S, Yedidia I (2016). Mol. Plant Pathol..

[36] Ngadze E, Icishahayo D, Coutinho TA, van der Waals JE (2012). Role of polyphenol oxidase, peroxidase, phenylalanine ammonia lyase, chlorogenic acid, and total soluble phenols in resistance of potatoes to soft rot. Plant Dis..

[37] Yan L (2013). Role of tomato lipoxygenase D in wound-induced jasmonate biosynthesis and plant immunity to insect herbivores. PLoS Genet..

[38] Zhou Y (2009). Over-expression of aspartate aminotransferase genes in rice resulted in altered nitrogen metabolism and increased amino acid content in seeds. Theor. Appl. Genet..

[39] Brauc S, De Vooght E, Claeys M, Höfte M, Angenon G (2011). Influence of over-expression of cytosolic aspartate aminotransferase on amino acid metabolism and defence responses against Botrytis cinerea infection in *Arabidopsis thaliana*. J. Plant Physiol..

[40] Kiefer IW, Slusarenko AJ (2003). The pattern of systemic acquired resistance induction within the Arabidopsis rosette in relation to the pattern of translocation. Plant Physiol..

[41] Villordon AQ (2009). Characterization of adventitious root development in sweetpotato. HortScience.

[42] Ruzin, S. E. *Plant Microtechnique and Microscopy*, Vol. 198 (Oxford University Press New York, 1999).

[43] Khadka N (2020). Host specificity and differential pathogenicity of Pectobacterium strains from dicot and monocot hosts. Microorganisms.

[44] Mead DA, Szczesna-Skorupa E, Kemper B (1986). Single-stranded DNA ‘blue’ T7 promoter plasmids: a versatile tandem promoter system for cloning and protein engineering. Protein Eng. Des. Sel..

