# Supplementary material for: Ecological adaptations influence the susceptibility of plants in the genus Zantedeschia to soft rot Pectobacterium spp
Source: Hortic Res. 2021 Jan 1;8:13. doi: 10.1038/s41438-020-00446-2 (PMC7775464; doi:10.1038/s41438-020-00446-2)
Supplement: Supplementary file 1 — Supplemental Material [file 41438_2020_446_MOESM1_ESM.docx]

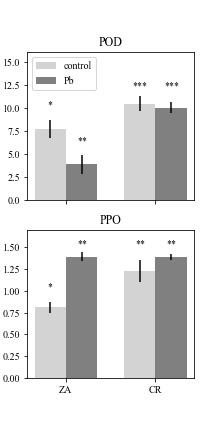


Figure S1: Peroxidase (POD) and polyphenol oxidase (PPO) activity of ZA and CR. Leaves were infiltrated at four points, each with 100µl distilled water (control) or *Pectobacterium brasiliense* suspension (10^6^ CFU/ml; treatment) and incubated for 48 hours at 28°C. Total proteins were extracted and activity was recorded with ABTS for POD and L-Dopa for PPO. Bars represent average enzymatic activity±SD of three replicates in a single experiment. Treatments labeled differently are significantly different (p<0.05).

**Method for Figure S1**

**Activity assays of oxidizing enzymes**

100mg leaf tissue 24 hours post infiltration with Pb or with distilled water (control) was frozen in liquid nitrogen and ground in a bead beater Mixer Mill (Retsch, Haan, Germany) with two 3-mm tungsten beads at 23 Hz/s for 2 minutes. The crashed leaf tissue was homogenized with 1ml of 10mM sodium acetate buffer (pH 5.6) with protease inhibitor (cOmplete, EDTA-free protease inhibitor cocktail, Roche diagnostics, Indianapolis, IN, USA) and centrifuged at 10,000g for 15 minutes at 4°C. The supernatant was used as crude extract. Protein concentration was determined according to Bradford assay with bovine serum albumin used as a standard. The activities of the oxidizing enzymes, peroxidases (PODs) and polyphenol oxidases (PPOs), were measured in protein extracts of ZA and CR. The assays were conducted with the following substrates: 2,2′-Azino-bis (3-ethylbenzothiazoline-6-sulfonic acid) di-ammonium salt (ABTS) for PODs activity and L-3,4-dihydroxyphenylalanine (L-Dopa) for PPOs activity. The reactions were performed in 96 well ELISA plates, with a mixture containing protein extract 25µl, substrate 50µl of 1mM ABTS or 5µl of 5mM DOPA (in10mM sodium acetate buffer, pH 5.6) and 25µl of 0.3% hydrogen peroxide. Total volume of each reaction was 100µl. The assay was monitored with a plate reader, Sepctra MR29010 (Dynex Tec, Chantilly, Virginia, USA) at 475 and 420nm for DOPA and ABTS respectively. The results were recorded for 60 minutes and expressed as OD∙min^-1^∙g^-1^ fresh weight.
