# Supplementary figures and images for: Ecological adaptations influence the susceptibility of plants in the genus Zantedeschia to soft rot Pectobacterium spp
Source: Hortic Res. 2021 Jan 1;8:13. doi: 10.1038/s41438-020-00446-2 (PMC7775464; doi:10.1038/s41438-020-00446-2)

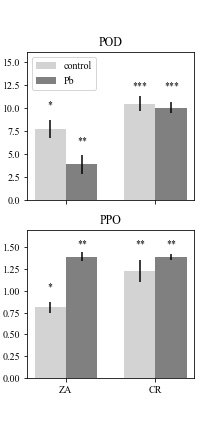

Supplement: Supplementary file 2 — Figure S1 [file 41438_2020_446_MOESM2_ESM.jpg]
